# Antimicrobial Resistance of *Clostridioides* (*Clostridium*) *difficile* in Cambodia

**DOI:** 10.3390/antibiotics14090950

**Published:** 2025-09-19

**Authors:** Lengsea Eng, Papanin Putsathit, Su-Chen Lim, Jessica M. Chisholm, Deirdre A. Collins, Archie C. A. Clements, Kefyalew Addis Alene, Thomas V. Riley

**Affiliations:** 1 School of Population Health, Faculty of Health Sciences, Curtin University, Bentley, WA 6102, Australia; 2 Laboratory Department, Calmette Hospital, Phnom Penh 120210, Cambodia; 3 Geospatial and Tuberculosis Research, The Kids Research Institute Australia, Nedlands, WA 6009, Australia; 4 School of Medical and Health Sciences, Edith Cowan University, Joondalup, WA 6027, Australia; 5 School of Biomedical Sciences, The University of Western Australia, Crawley, WA 6009, Australia; 6 PathWest Laboratory Medicine, Ellenbrook, WA 6069, Australia

**Keywords:** *Clostridioides difficile*, Cambodia, antimicrobial resistance, metronidazole, vancomycin, fidaxomicin, rifaximin

## Abstract

**Background/Objectives:** Antimicrobial resistance (AMR) remains a major topic of interest in infectious disease management. We studied AMR in *Clostridioides difficile* isolated in Cambodia. **Methods:** Agar dilution susceptibility testing was performed according to the CLSI guidelines to determine minimal inhibitory concentrations (MICs) of 10 antimicrobials for 192 isolates of *C. difficile* from four populations in Cambodia: hospitalised adults, hospitalised children, children from an outpatient department (OPD), and healthy adolescents in the community. **Results:** Using the CLSI MIC breakpoints for anaerobes and EUCAST breakpoints for *C. difficile*, all isolates were susceptible to vancomycin, metronidazole, fidaxomicin, and amoxicillin/clavulanic acid, and none were resistant to meropenem. The resistance proportions were for clindamycin, 88% (169/192); tetracycline, 50% (96/192); moxifloxacin, 20% (38/192); and rifaximin, 8% (15/192). Among the 169 clindamycin-resistant isolates, 56.8% (96/169) had an erythromycin MIC of >512 mg/L. Multidrug resistance (MDR) occurred in 20% (39/192) of the isolates, of which 82% (32/39) were non-toxigenic strains. The proportion of MDR varied between collections of isolates from different populations: 28.6% (22/77) in hospitalised adults, 29.8% (14/47) in hospitalised children, 5% (3/59) in OPD children, and none (00/07) in healthy adolescents in the community. **Conclusions:** *C. difficile* isolates from Cambodia remained susceptible to antimicrobials used to treat *C. difficile* infection: vancomycin, metronidazole, and fidaxomicin; however, high proportions of resistance to clindamycin and tetracycline were observed. The high number of MDR strains of *C. difficile* is a threat to AMR management in Cambodia and a factor contributing to the persistent spread of *C. difficile* in both hospital and community settings.

## 1. Introduction

Antimicrobial resistance (AMR) remains an important issue worldwide, and high mortality rates have been reported attributable to and associated with bacterial AMR [1,2]. *Clostridioides difficile* infection (CDI) is driven by the same factor that drives AMR: antimicrobial use. Thus, in 2013, *C. difficile* was thought of as a significant AMR pathogen and an “immediate public health threat that requires urgent and aggressive action” by the US CDC [2]. While the treatment of CDI is challenging due to the high number of recurrent infections (13–45%) [3,4], AMR presents additional challenges to CDI patient management. Since *C. difficile* can colonise humans and animals intermittently and exists in the environment, antimicrobial usage to treat non-*C. difficile* infection in humans and animals, or to improve animal growth, and other uses in agriculture could potentially induce resistance in *C. difficile* present in the same setting [5,6].

Fidaxomicin, vancomycin, and rifaximin are the treatments recommended in CDI management [7,8]. Metronidazole is no longer recommended as first-line therapy, although it continues to be used in many countries [9,10] and remains a recommendation as second-line therapy or in the absence of first-line treatment [7,8]. Faecal microbial transplantation and surgery are recommended for recurrent CDI and severe complicated CDI, respectively. Besides these challenges in the management of primary and recurrent CDI, AMR in *C. difficile* has been reported at varying rates by country, study year, or genotype [11,12,13]. Resistance of *C. difficile* to metronidazole and vancomycin has been reported at up to 2.7% and 14.3%, respectively [12,14,15,16,17,18,19]; however, much less to fidaxomicin [20,21]. In Asia, metronidazole remains the treatment of choice due to its efficacy and low cost, and the unavailability of other drugs like oral vancomycin and fidaxomicin [9,12]. Resistance to other agents, particularly the macrolide lincosamide streptogramin B (MLS_B_) group, tetracycline, and moxifloxacin, occurs commonly—up to 90%, 30%, and 44.4%, respectively [11,12,17,18,19]. In addition, *C. difficile* acquires resistance genes relatively easily from mobile genetic elements (MGEs), often transposons commonly carrying resistance determinants [22,23,24].

AMR in *C. difficile* has been studied in many countries where CDI is recognised, but not much in low- to middle-income countries like Cambodia. This study aimed to investigate AMR of *C. difficile* isolates from community and hospital settings in Cambodia.

## 2. Results

### 2.1. Antimicrobial Resistance

**Figure 1:**
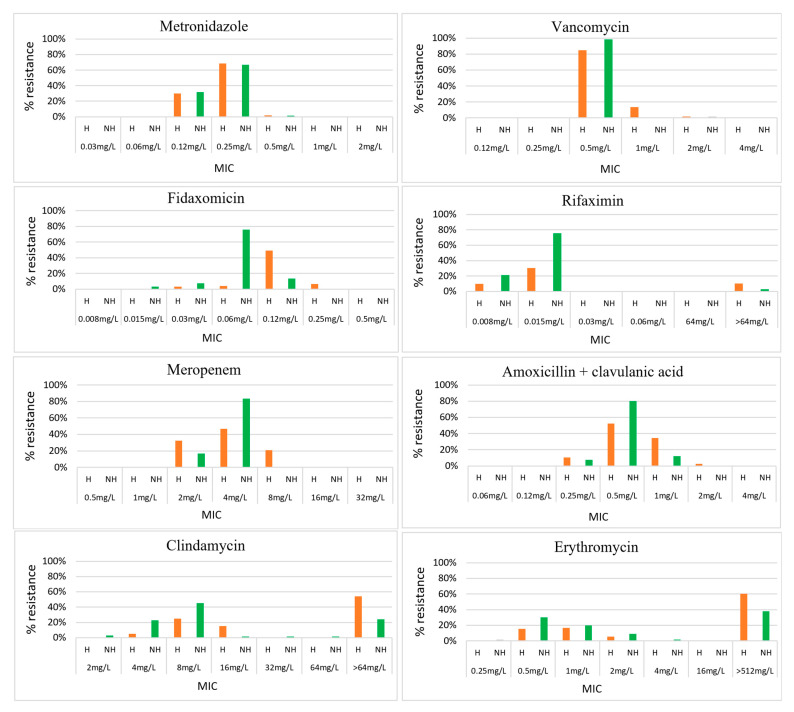

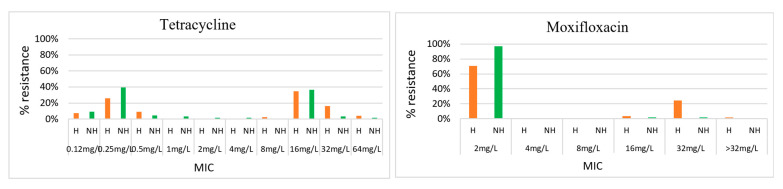
Minimal inhibitory concentrations for 10 antimicrobials against 124 *C. difficile* isolates from hospitalised patients and 66 *C. difficile* isolates from non-hospitalised individuals in Cambodia. H: hospitalised patients, in orange; NH: non-hospitalised individuals, in green; MIC: minimum inhibitory concentration.

According to the minimal inhibitory concentration (MIC) breakpoints of the CLSI for anaerobes and EUCAST for *C. difficile*, all isolates were susceptible to vancomycin, metronidazole, fidaxomicin, and amoxicillin/clavulanic acid (Table 1). Of the 192 isolates, 98% were inhibited by vancomycin at 1 mg/L and metronidazole at 0.25 mg/L, while 96% were inhibited by fidaxomicin at 0.12 mg/L (Figure 1). According to MIC breakpoints of the CLSI for anaerobes, 88% (169/192), 50% (96/192), 20% (38/192), and 8% (15/192) of all isolates were resistant to clindamycin, tetracycline, moxifloxacin, and rifaximin, respectively (Table 1). Both the EUCAST and CLSI guidelines had no interpretive MIC breakpoints for erythromycin; however, erythromycin had a high MIC50/MIC90 of >512 mg/L/>512 mg/L, and 52% of isolates had an MIC > 512 mg/L. Among clindamycin-resistant *C. difficile*, 56.8% (96/169) also had an erythromycin MIC > 512 mg/L.

Multidrug resistance (MDR), resistance to at least three antimicrobial categories [25], occurred in 20% (39/192) of all isolates, mostly in *C. difficile* RTs QX011 (*n* = 22), QX002 (*n* = 3), QX712 (*n* = 3), QX710 (*n* = 2), 046 (*n* = 2), and one isolate each of seven other RTs. Non-toxigenic strains accounted for 82% (32/39) of MDR strains. The proportions of MDR strains varied greatly between different population groups: 28.6% (22/77) in hospitalised adults and 29.8% (14/47) in hospitalised children versus 5% (3/59) in OPD children and none in healthy adolescents in the community. Resistance to clindamycin, tetracycline, and moxifloxacin occurred in 82% (32/39) of MDR cases.

### 2.2. Antimicrobial Resistance by Condition

**Figure 2:**
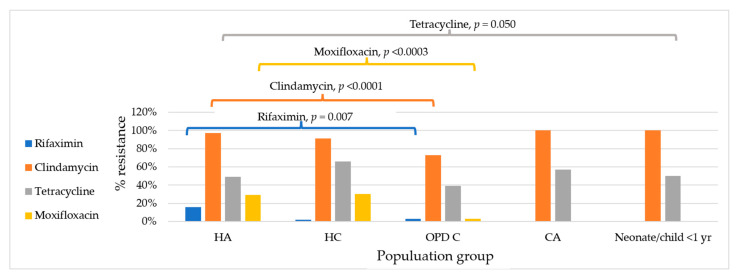
Antimicrobial resistance in *C. difficile* by population group. HA: hospitalised adults; HC: hospitalised children; OPD C: children visiting an outpatient department; CA: healthy adolescents in the community.

**Figure 3:**
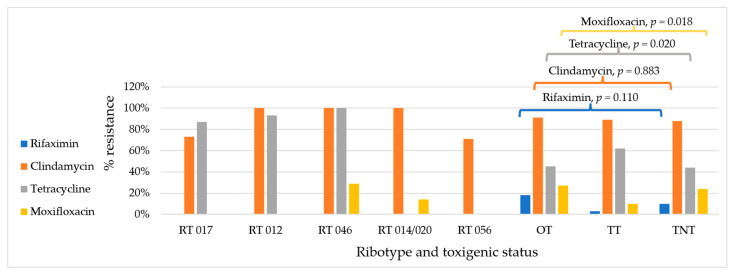
Antimicrobial resistance in *C. difficile* by ribotype and toxigenic status. OT: other toxigenic strains or all toxigenic strains excluding RTs 017, 012, 046, 014/020, and 056 (*n* = 11); TT: total toxigenic strains or all strains that contained at least one toxin gene or any combination (*n* = 61); TNT: total non-toxigenic strains or all stains that did not contain any toxin genes (*n* = 131).

When comparing hospitalised patients to non-hospitalised individuals, *C. difficile* resistance to clindamycin (95% vs. 74%, *p* ≤ 0.0001) and moxifloxacin (29% vs. 3%, *p* ≤ 0.0001) was significantly higher in hospitalised patients than in non-hospitalised individuals (Table 2 and Figure 1). Resistance to rifaximin was more common in hospitalised adults (Figure 2). By residency of cases, up to 10% of isolates from those living outside the capital city were resistant to rifaximin versus 0% in those living in the capital city, while resistance to tetracycline was greater in isolates from those living in the capital city, 68% vs. 46% (*p* = 0.020) (Table 2). By toxigenic status, non-toxigenic isolates from hospitalised patients were more resistant to moxifloxacin than toxigenic strains (35% vs. 14%, *p* = 0.017), and toxigenic isolates from non-hospitalised patients were more resistant to tetracycline than non-toxigenic strains (67% vs. 26%, *p* = 0.001) (Table 3 and Figure 3). Among toxigenic strains, rifaximin resistance (18%, 11/61) was seen only in unknown and uncommon toxigenic strains (Figure 3). While none of RT 046 were susceptible to tetracycline and clindamycin, and none of RT 012 were susceptible to clindamycin, 93% of them were resistant to tetracycline, and only 87% and 73% of RT 017 were resistant to tetracycline and clindamycin, respectively (Figure 3).

## 3. Discussion

This research aimed to study the antimicrobial susceptibility of *C. difficile* isolates from Cambodia. While some of the findings were anticipated, the high number of MDR strains of *C. difficile* was not and is a threat to AMR management in Cambodia. This may be a factor contributing to the persistent spread of *C. difficile* in both hospital and community settings. The significant difference in clindamycin and moxifloxacin resistance in *C. difficile* isolates from hospitalised patients versus non-hospitalised individuals strongly suggests antimicrobial selective pressure in hospitals on bacteria residing as commensals or saprophytes and facilitated by higher antimicrobial consumption in hospitalised patients. Another possibility is the transmission of resistance determinants between bacteria pre-existing in hospital settings and *C. difficile* from the community, brought into hospital settings by the patients.

MDR is defined as resistance to at least three antimicrobial categories [25], and, if more antimicrobials were tested, it is possible that there would be more MDR strains. In the current study, 10 antimicrobial classes were tested, including agents for treatment and CDI-inciting agents. The overall prevalence of MDR (20%, 39/192) in the collection of *C. difficile* strains from different population groups in Cambodia is comparable to the results in a study conducted in Thailand (21.9%, 23/105) in 2015 [26], but more than twice the prevalence (9.03%, 29/321) in another study from the same hospital in Thailand in 2017–2018 [17]. Noticeably, Vietnam had a similar proportion of MDR strains found in diarrhoeic adults (27.3%, 9/33) [18]. These findings do not directly reflect the different resistance patterns of *C. difficile* in the three countries due to different study participants; however, similarities in antimicrobial consumption in individual hospitals could be assumed. Although there was a high prevalence of MDR, all the isolates were susceptible to antimicrobials for CDI treatment, with low MICs, except rifaximin (8% were resistant).

*C. difficile* becomes resistant to rifaximin and rifampin by mutations in RpoB, the β subunit of the RNA polymerase for protein synthesis [27]. Rifaximin-resistant *C. difficile* in the current study was found only in patients living outside the capital city, and only in unknown and novel toxigenic strains, suggesting that resistance to rifaximin is more likely to occur in community-associated local strains. The prevalence of rifaximin resistance in the current study (8%) was between that found in neighbouring countries, Thailand (10%) [17] and Vietnam (5%) [18], and very similar to North America (7.9–12.4%) [14] and some countries in Europe (0–19.2%) [16,28]. Rifaximin, a derivative of rifampin, is not widely available in Cambodia, but rifampin is a drug used in the first-line treatment of tuberculosis (TB) in the country. Cambodia has endemic TB, with an incidence of 320 per 100,000 population reported in 2022, according to the Tuberculosis Report 2023 by the Ministry of Health of Cambodia [29]. The long-term use of rifampin in patients with TB could be a reason for the high resistance to rifaximin in *C. difficile* in this country. This is also a reason why patients with TB could acquire *C. difficile* and develop CDI during treatment of TB, while rifaximin-susceptible cases could be treated with the ongoing rifampin treatment for TB [30,31], and other treatments for CDI besides rifampin might be needed in resistant cases [9].

Tetracycline resistance commonly derives from resistance genes such as *tetM*, *tet40*, and *tet44* acquired via MGEs, preventing the binding of tetracycline to 16S rRNA [22]. Though MGEs were similar between toxigenic and non-toxigenic strains [24], the significant difference in resistance between toxigenic and non-toxigenic strains in the current study suggests other possible non-genetic determining factors/mechanisms in toxigenic and non-toxigenic *C. difficile* need to be investigated for resistance to tetracycline. Tetracycline resistance in *C. difficile* RT 078 driven by selective pressure has been reported as possibly having a role in the spread of this strain, with up to 76.5% possessing the *tetM* gene [32]. Although there were no *C. difficile* RT 078 strains isolated in the current study, selective pressure on other strains of *C. difficile* could induce tetracycline resistance and spread because of tetracycline use in agriculture. In an earlier study of CDI in Cambodia, it was reported that patients might carry *C. difficile* before being admitted to the hospitals [33]. Those patients were all asymptomatic and all from provinces where agricultural activities were common, suggesting some *C. difficile* isolates found in hospital settings might be acquired from areas with agricultural activities. Tetracycline is used in humans and animals for disease prevention and treatment and was used for animal growth promotion [6]. Resistance to tetracycline in the current study in Cambodia requires further investigation. The poor knowledge of food producers in Cambodia regarding antimicrobial use in livestock is a potential factor contributing to the increase in AMR in Cambodia in general and particularly in *C. difficile*, where a lack of awareness of CDI is already a concern [34].

The spread of *C. difficile* RT 027 in North America [35] and Europe [16,36] in the early 2000s was driven by the high consumption of fluoroquinolones. Resistance to fluoroquinolones derives from point substitutions in GyrA and/or GyrB subunits of DNA gyrase, reducing the binding of antimicrobials to the target [22]. The prevalence of moxifloxacin resistance in the current study (20%) was comparable to that in Thailand (24%) [17] and in the USA (17.5–28.7%) [14], but much less than a previous study in the Asia-Pacific region (44.4%) [12] and in Europe (57.1%) [16]. The reasons for these differences are not known but may relate to variations in fluoroquinolone usage and the diversity of *C. difficile* genotypes in each country.

Resistance to clindamycin and erythromycin is commonly present in *C. difficile* from the Asia-Pacific region, compared to other tested antimicrobial classes [12,15]. The resistance mechanism is by methylating 23S rRNA, preventing the binding of antimicrobials, resulting in a high level of resistance to the MLS_B_ drugs, clindamycin and erythromycin. The results from the current study are comparable to previous studies in Asia [12,17,19], suggesting high consumption of MLS_B_ antimicrobials in the region. *C. difficile* becomes resistant to MLS_B_ by integrating MGEs containing resistance determinants, though unknown mechanisms have been frequently suggested [24]. The resistance derives from the acquisition of *erm* class genes, more commonly *ermB*, *erm(52)*, and *ermG*; non-*erm* genes, *mefH* and *mefA*; and *msrD* [22]. In earlier studies in Cambodia, individuals in hospital and community settings were frequently colonised by *C. difficile* asymptomatically, and there was no proper diagnosis [33]. Thus, with high resistance to MLS_B_ drugs, *C. difficile* might be a silent vector transmitting AMR genes in the region, particularly the *erm* class genes.

Non-toxigenic *C. difficile* was resistant to more classes of antimicrobials than toxigenic strains in this study (Figure 3). This emphasises the importance of gaining knowledge about all strains of *C. difficile* in Asia, not just toxigenic strains causing CDI in the region. Asian strains of *C. difficile* are largely non-toxigenic or produce only toxin B, such as *C. difficile* RT 017, which has caused outbreaks worldwide [37,38]. Noticeably, though RT 017 is less resistant to clindamycin and tetracycline (Figure 3), compared to RTs 012 and 046, RT 017 remains predominant in hospital settings. This suggests that resistance to different antimicrobial classes is not the only parameter determining the epidemiology of CDI in each setting.

There is only one paper from Cambodia that described some environmental contamination, and that was contaminated food [39]. Other possible sources and reservoirs of *C. difficile* in the environment were discussed in three earlier studies from our group [33,40,41]. *C. difficile* environmental contamination has also been described in studies in neighbouring countries, e.g., Vietnam [42], which is similar to Cambodia. The AMR characteristics of *C. difficile* in the current study could imply the usage of antimicrobials in different ways in Cambodia, not just for the treatment of infectious diseases. Further investigation into sources/reservoirs of *C. difficile* in relation to its AMR is needed.

There are some limitations in this study. First, all the strains were from mostly asymptomatic carriers, as described in the previous studies [33,40,41]; thus, the findings from this study only represent the antimicrobial susceptibility of circulating strains in the country and not isolates from cases of CDI. Deciding how these results will impact the treatment for CDI in this country requires further work. Second, the mechanisms of resistance were not investigated, making it difficult to correlate resistance with possible drivers of resistance; however, this topic will be the basis for a future publication. The application of whole-genome sequencing will allow the identification of resistance genes and possibly reflect antimicrobial usage in the country, adding value to AMR surveillance.

## 4. Materials and Methods

### 4.1. C. difficile Isolates

In total, 192 isolates were included in this study: 63 isolates from hospitalised adults [33], 47 isolates from hospitalised children [40], 59 isolates from children visiting an outpatient department, 07 isolates from healthy adolescents, 14 isolates from an unpublished pilot study of CDI in hospitalised adults, and two isolates (one from a neonate and one from a child <1 year old) from another unpublished Cambodian study. The majority of isolates, 90% (173/192), were recovered from non-diarrheal, normal, and hard stools.

Of the 192 isolates, 68% (131/192) were non-toxigenic. Among the toxigenic strains, *C. difficile* ribotypes (RTs) 017 (8%, 15/192) and 012 (7%, 14/192) were the two most common, while RTs 046, 014/020, and 056 were equally the third most common (Table 4). Among the 61 toxigenic strains, *C. difficile* with the toxin profile A+B+CDT- accounted for 69% (42/61); A-B+CDT-, 29% (18/61); and A+B+CDT+, 2% (1/61).

### 4.2. MIC Determination

The cryopreserved isolates were recovered on horse blood agar (BA), incubated in an A35 anaerobic chamber (Don Whitley Scientific, Ltd., Shipley, West Yorkshire, UK) at 35 °C for 48 h, with an atmosphere of 80% N_2_, 10% CO_2_, and 10% H_2_ and a 75% relative humidity. A second subculture was conducted to confirm purity before testing. MIC determination was performed using the agar incorporation method according to the CLSI guidelines for anaerobic bacteria (M11-A7) [43]. Antimicrobials for the treatment of CDI, including metronidazole, vancomycin, fidaxomicin, and rifaximin; those with a high risk of inducing CDI, including clindamycin, erythromycin, and moxifloxacin; and those with a variable risk for CDI, including amoxicillin/clavulanic acid, meropenem, and tetracycline, were chosen for testing. The interpretation of rifaximin susceptibility followed O’Connor et al. [27]. Breakpoints for metronidazole, vancomycin, and fidaxomicin were those in the EUCAST guidelines for *C. difficile* [44] and, for other antimicrobials, the recommendations in the CLSI guidelines were followed [45].

### 4.3. Statistical Analysis

Summary statistics for categorical variables were reported as proportions. A Chi-squared test was conducted to examine associations between the resistance and ribotype, toxigenic status, host, and setting. A *p*-value < 0.05 was chosen for statistical significance.

## 5. Conclusions

To the best of our knowledge, this is the first study of AMR in *C. difficile* from different population groups in Cambodia. Despite the high number of MDR strains, all *C. difficile* isolates remained susceptible to CDI treatment drugs, including vancomycin, metronidazole, and fidaxomicin. The resistance patterns of *C. difficile* necessitate improved infection prevention and control of *C. difficile* in the country, though AMR is not the only determining factor in the persistence of *C. difficile* in each setting.

## Figures and Tables

**Table 1:** Antimicrobial susceptibility of 192 Cambodian strains of *C. difficile*.

Antimicrobial	Interpretive Categories and MIC Breakpoints (mg/L)			Result Ranges (mg/L)	MIC50/MIC90 (mg/L)	Susceptibility % (N = 192)		
	S	I	R			S	I	R
Vancomycin	≤2		>2	0.5–2	0.5/1	100%	0	0
Metronidazole	≤2		>2	0.12–0.5	0.25/0.25	100%	0	0
Fidaxomicin	≤0.5	-	>0.5	0.015–0.25	0.06/0.12	100%	0	0
Rifaximin	<32		≥32	0.0008–>64	0.015/0.015	92% (177)	0	8% (15)
Meropenem	≤4	8	≥16	2 to 8	4/8.	86% (166)	14% (26)	0
Amoxicillin/clavulanic acid	≤4	8	≥16	0.25–2	0.5/1	100%	0	0
Clindamycin	≤2	4	≥8	2–>64	16/>64	1% (1)	11% (21)	88% (169)
Erythromycin	-		-	0.25–>512	>512/>512	-	-	-
Tetracycline	≤4	8	≥16	0.12–64	8/32	48%	2% (3)	50% (96)
Moxifloxacin	≤2	4	≥8	2–>32	2/32	80%	0	20% (38)

**Table 2:** Antimicrobial resistance of *C. difficile* by hospitalisation status and host residency.

Antimicrobials	Interpretive Categories and MIC Breakpoints (mg/L)			*C. difficile* in Hospitalised Patients (*n* = 124)	*C. difficile* in Non-Hospitalised Individuals (*n* = 66)	*p*-Value	*C. difficile* in Those Living in the Capital City (*n* = 37)	*C. difficile* in Those Living Outside the Capital City (*n* = 145)	*p*-Value
	S	I	R	Resistant (%)	Resistant (%)		Resistant (%)	Resistant (%)	
Vancomycin	≤2		>2	0%	0%		0%	0%	
Metronidazole	≤2		>2	0%	0%		0%	0%	
Fidaxomicin	≤0.5	-	>0.5	0%	0%		0%	0%	
Rifaximin	<32		≥32	10%	3%	0.07	0%	10%	
Meropenem	≤4	8	≥16	0%	0%		0%	0%	
Amoxicillin/clavulanic acid	≤4	8	≥16	0%	0%		0%	0%	
Clindamycin	≤2	4	≥8	95%	74%	<0.0001	92%	88%	0.464
Erythromycin	-		-	-	-		-	-	
Tetracycline	≤4	8	≥16	55%	41%	0.067	68%	46%	0.020
Moxifloxacin	≤2	4	≥8	29%	3%	<0.0001	24%	18%	0.378

**Table 3:** Antimicrobial resistance of *C. difficile* by toxigenic status.

Antimicrobials	Interpretive Categories and MIC Breakpoints (mg/L)			Hospitalised Patients			Non-Hospitalised Individuals		
				Toxigenic Strains (*n* = 36)	Non-Toxigenic Strains (*n* = 88)	*p-*Value	Toxigenic Strains (*n* = 24)	Non-Toxigenic Strains (*n* = 42)	*p-*Value
	S	I	R	Resistant (%)	Resistant (%)		Resistant (%)	Resistant (%)	
Vancomycin	≤2		>2	0%	0%		0%	0%	
Metronidazole	≤2		>2	0%	0%		0%	0%	
Fidaxomicin	≤0.5	-	>0.5	0%	0%		0%	0%	
Rifaximin	<32		≥32	3%	14%	0.073	4%	2%	0.684
Meropenem	≤4	8	≥16	0%	0%		0%	0%	
Amoxicillin/clavulanic acid	≤4	8	≥16	0%	0%		0%	0%	
Clindamycin	≤2	4	≥8	94%	95%	0.812	79%	71%	0.489
Erythromycin	-		-	-	-		-	-	
Tetracycline	≤4	8	≥16	58%	53%	0.617	67%	26%	0.001
Moxifloxacin	≤2	4	≥8	14%	35%	0.017	4%	2%	0.684

**Table 4:** Number of *C. difficile* ribotypes in each population group.

Ribotype	Hospitalised Adults	Hospitalised Children	OPD Children	Adolescents	Neonate and Children < 1 yr	Total
017	5	1	7	1	1	15
012	2	7	2	3	0	14
046	5	0	2	0	0	7
014/020	3	3	1	0	0	7
056	3	0	3	1	0	7
Other toxigenic strains	6	1	2	2	0	11
Non-toxigenic strains	53	35	42	0	1	131
**Total**	**77**	**47**	**59**	**7**	**2**	**192**

## Data Availability

The data presented in this study are available on request from the corresponding author.

## Abbreviations

The following abbreviations are used in this manuscript:

AMR	Antimicrobial resistance
CDI	*Clostridioides difficile* infection
MLS_B_	Macrolide lincosamide streptogramin B
MGEs	Mobile genetic elements
MIC	Minimal inhibitory concentration
BA	Blood agar
RT	Ribotype
MDR	Multidrug resistance
OPD	Outpatient department
S	Susceptible
I	Intermediate
R	Resistant
TT	Total toxigenic strains
OT	Other toxigenic strains
TNT	Total non-toxigenic strains
